# Chronic Exposure to Cadmium Disrupts the Adrenal Gland Activity of the Newt *Triturus carnifex* (Amphibia, Urodela)

**DOI:** 10.1155/2013/424358

**Published:** 2013-07-22

**Authors:** Flaminia Gay, Vincenza Laforgia, Ivana Caputo, Carla Esposito, Marilena Lepretti, Anna Capaldo

**Affiliations:** ^1^Department of Biology, University Federico II, Via Mezzocannone 8, 80134 Naples, Italy; ^2^Department of Chemistry and Biology, University of Salerno, Fisciano, 84084 Salerno, Italy

## Abstract

We intended to verify the safety of the freshwater values established for cadmium by the European Community and the Italian Ministry of Health in drinking water (5 **μ**g/L) and sewage waters (20 **μ**g/L). Therefore, we chronically exposed the newt *Triturus carnifex* to 5 **μ**g/L and 20 **μ**g/L doses of cadmium, respectively, during 3 and 9 months and verified the effects on the adrenal gland. We evaluated the serum concentrations of adrenocorticotropic hormone (ACTH), corticosterone, aldosterone, norepinephrine, and epinephrine. During the 3-month exposure, both doses of cadmium decreased ACTH and corticosterone serum levels and increased aldosterone and epinephrine serum levels. During the 9-month exposure, the 5 **μ**g/L dose decreased ACTH and increased aldosterone and epinephrine serum levels; the 20 **μ**g/L dose decreased norepinephrine and epinephrine serum levels, without affecting the other hormones. It was concluded that (1) chronic exposure to the safety values established for cadmium disrupted the adrenal gland activity and (2) the effects of cadmium were related both to the length of exposure and the dose administered. Moreover, our results suggest probable risks to human health, due to the use of water contaminated by cadmium.

## 1. Introduction

Cadmium (Cd) is a naturally ubiquitous heavy metal, very toxic to aquatic and terrestrial wildlife, with no known biological function [1]. Cadmium enters the aquatic systems from industrial and consumer waste contaminating the aquatic environment; it has an extremely long biological half-life (about 30 years) in both humans and experimental animals [2] and accumulates in vertebrates throughout the food chain [3], damaging the physiological processes or tissues in aquatic organisms even at concentrations far below the lethal level [4]. 

Cadmium exerts a large number of adverse effects on ecosystems and human and animal health [5, 6]; this metal is embryotoxic, causing different kinds of malformations and lethality in mammals [7, 8] as well as amphibians [1, 9–14]. In fish, there is substantial evidence that growth, reproduction, respiratory functions, and osmoregulation are affected by exposure to cadmium [15–17]. In amphibians, cadmium is genotoxic [18, 19]; it accumulates in peripheral tissues as liver and kidney [3, 20] and induces liver and kidney toxicity [2, 21]. Cadmium behaves like an endocrine disruptor, exerting oestrogenic as well as androgenic effects in various species including fish [22–25]. Recently, it has also been reported that chronic exposure to cadmium depresses in fish the hypothalamic-pituitary-adrenocortical axis and impairs cortisol secretion [17, 26–28], whereas almost no research has been conducted on the effects of cadmium on the endocrine system in amphibians. 

In freshwater, cadmium concentration ranges from 2 to 30 *μ*g/L [29]. The European Community [30] and the Italian Ministry of Health [31] have established a freshwater safety value for cadmium of 5 *μ*g/L in drinking water and 20 *μ*g/L in sewage waters. However, considering the toxicity of this heavy metal to a wide variety of organisms and its persistence in ecosystems, we wondered whether these doses were really safe for human and animal health. To this aim, we chose an urodele amphibian, the newt *Triturus carnifex*, for the experimental model and verified the effects of a chronic exposure to cadmium concentrations of 5 *μ*g/L and 20 *μ*g/L, respectively, on the adrenal gland, on the ground of the following considerations. First of all, the urodele amphibians spend a great part of their life in the aquatic environment and therefore can easily adsorb waterborne chemicals through their permeable skin; indeed, aquatic contamination is believed to have reduced amphibian abundance and species richness at many sites [13]. In addition, *T. carnifex* has the features of an ideal bioindicator, due to its high sensitivity to chemicals [32, 33]. 

In the second place, the amphibian adrenal gland plays a key role in the stress response, which includes the release of both corticosteroids and catecholamines. Corticosterone and, to a lesser extent, aldosterone are gluconeogenic and hyperglycaemic, but the primary role of these steroids appears to be the regulation of ion and water balance. Epinephrine is a potent stimulator of lipolysis in fat bodies and of glycogenolysis in both liver and muscle and is therefore hyperglycemic. These hormones induce changes in metabolism and/or ionic regulation that work to combat physiological factors and to eliminate or to neutralize the stressful stimulus, allowing the organism to adapt to its environment and to survive [34]; moreover, the adrenal gland is involved in the amphibian reproductive processes [35]. Complex neuroendocrine mechanisms are involved in the regulation of the amphibian adrenal gland. Adrenal corticosteroid secretion is stimulated by adrenocorticotropic hormone (ACTH, which in turn is controlled by corticotropin-releasing hormone (CRH)), arginine vasotocin (AVT), and renin-angiotensin system; moreover, natriuretic peptides inhibit the actions of ACTH [34]. In the newt *Triturus carnifex*, the steroidogenic tissue mainly produces the corticosteroids aldosterone and corticosterone; the chromaffin tissue has only a type of chromaffin cell, producing the catecholamines, norepinephrine, and epinephrine in accordance with a functional cycle, and correlated with the environmental temperature. During both the December–February and May–August periods, large quantities of norepinephrine and small quantities of epinephrine granules are present in the chromaffin cells. During the March-April and September–November periods, the production of epinephrine increases and the chromaffin cells contain almost the same quantities of norepinephrine and epinephrine granules [36, 37]. Both corticosteroids and catecholamines show an annual and a daily pattern, related to the metabolic activity of this species [37–39]. Moreover, the adrenal gland of *T. carnifex* exhibits paracrine relationships between the steroidogenic and the chromaffin tissues since the steroidogenic tissue modulates the activity of the chromaffin tissue, stimulating the release of catecholamines, and the chromaffin tissue modulates the activity of the steroidogenic tissue, increasing (norepinephrine) or decreasing (epinephrine and dopamine) the release of corticosteroids [40]. 

## 2. Materials and Methods

### 2.1. Reagents

Cadmium chloride (CdCl_2_, CAS no. 10108-64-2, ≥99.0%) was obtained from Sigma-Aldrich (St. Louis, MO 63103, USA). A stock solution of CdCl_2_ was prepared in distilled water at a concentration of 0.08 g/L.

### 2.2. Animals

Adult male specimens of *T. carnifex* (mean mass 8.0 g), captured in the field around Naples, were kept in 50 L glass tanks (10 newts per tank, with a loading rate of 1.6 g newt/L water), under a natural photoperiod, in dechlorinated, well-aerated tap water, at seasonal temperature. The animals were fed minced cow liver and used after an acclimation period of 2 weeks.

### 2.3. Experimental Design

At the beginning of February, 60 adult male newts were divided into 6 groups, each containing 10 specimens. The first 2 groups were exposed daily to 5 *μ*g/L and 20 *μ*g/L doses, respectively, of CdCl_2_; the exposure lasted 3 months and ended at the beginning of May. The 3rd and the 4th groups were exposed daily to 5 *μ*g/L and 20 *μ*g/L doses, respectively, of CdCl_2_; the exposure lasted 9 months and ended at the beginning of November. Two control groups, one for each of the 2 different lengths of exposure, were exposed to tap water only. The water was changed every day in all the tanks. The test was carried out in triplicate. At the beginning of May (3-month exposure) and November (9-month exposure) the newts were anaesthetized by hypothermia, chilling them in chipped ice, within 5 min after removal from tank. Blood was immediately collected over 3 min by heart puncture, between 11 AM and 2 PM, centrifuged for 15 min at 2,000 g, and serum was collected and stored, together with standards, to correct for degradation, at –22°C until assayed. Institutional committees (department of health) approved the experiments, which were organized to minimize the stress and the number of animals used.

### 2.4. Hormone Assay

Aldosterone and corticosterone serum levels were determined by radioimmunoassay (RIA) as previously described [32, 33]. 

Briefly, nonhemolyzed serum samples (80 *μ*L for aldosterone and 30 to 40 *μ*L for corticosterone) were incubated for 30 min at 37°C with known amounts of radioactive steroids (^3^H-aldosterone and ^3^H-corticosterone from Bio-Rad, Hercules, CA, USA) in 0.06 M Na-phosphate buffer containing 0.01 M EDTA disodium salt and 0.1% BSA pH 7.4. Samples were applied to an extraction column (Sep-Pak C18, Waters, Milford, MA, USA) and washed with 500 *μ*L of pure methanol. Methanol extracts were dried at 37° under vacuum and redissolved in 1,400 *μ*L of PBS. An aliquot was taken to determine the labeled hormone recovery, and on two other aliquots aldosterone and corticosterone were assayed by RIA. After incubation with rabbit antiserum (Biogenesis, Poole, UK) for 30 min at 37°C and for another 2 h in an ice bath, dextran-coated charcoal was used to separate free from bound steroids. After immersion for 10 min in an ice bath and centrifugation (2,000 rpm), a supernatant aliquot was counted with a liquid scintillation spectrometer (Tri-Carb Packard, GMI, Albertville, MN, USA). Extraction yields ranged from 80% to 90% for both hormones. Data were obtained through a standard calibration curve linearized with a log-logit method and corrected for individual extraction yield. Sensitivity was 5 pg/tube for aldosterone and corticosterone. Intra-assay coefficient of variation was 10% and interassay coefficient of variation was 12% for both steroids.

ACTH concentrations were measured in 100 *μ*L of plasma by a two-site immunoradiometric assay using mouse monoclonal antibodies (Diagnostic Products Corp.) as previously described [33]. Cross-reactivities of the ACTH antiserum as determined by the kit manufacturer were 0.03% for a-MSH, 0.01% for *β*-MSH, and 0.02 for *β*-endorphin. Sensitivity was 0.1 pg · mL^−1^ as determined by the kit manufacturer, and the inter- and intra-assay coefficients of variation were 10% and 6%, respectively.

 Norepinephrine and epinephrine levels were determined in 150 *μ*L serum. For catecholamine extraction, 50 *μ*L of dihydroxybenzylamine was added as an internal standard. Ten milligrams activated aluminum oxide (Sigma, St. Louis, MO, USA) was used as adsorbent for catecholamines and the internal standard. After 15 min shaking and centrifugation, the supernatant was removed and the aluminum oxide containing the adsorbed catecholamines and the internal standard was washed three times with 1 mL distilled water by shaking, centrifuging, and discarding the supernatant-extracted samples using high-performance liquid chromatography (HPLC), with electrochemical detection, according to the method previously used in *Triturus carnifex* [32, 33]. Electrochemical HPLC detection was carried out using an acid eluant; NE and E levels were calculated in comparison to the internal standard (dihydroxybenzylamine). The detection limit for NE and E was around 20 pg.

### 2.5. Statistical Analysis

The values were expressed as means ± standard error of mean (SEM). All the data were first tested for normality and homogeneity of variance to meet statistical demands; the homogeneity of variance was assessed by the Bartlett test. The data were compared by one-way analysis of variance (ANOVA), followed by the Tukey-Kramer multiple comparison test. Differences were considered significant when *P* < 0.05.

## 3. Results

**Figure 1:**
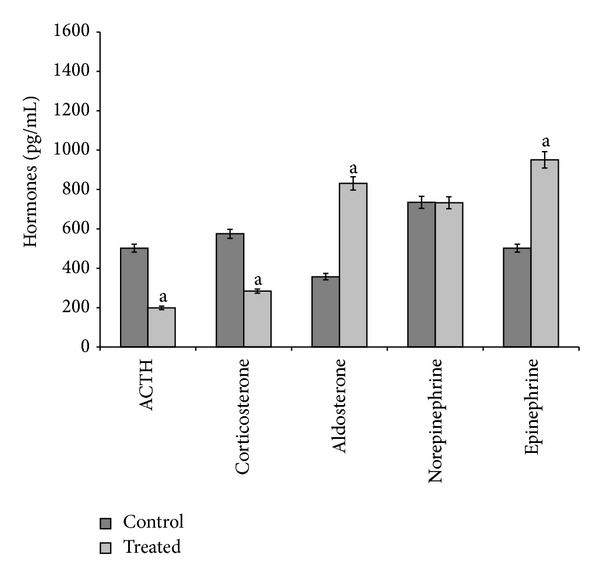
ACTH, corticosterone, aldosterone, norepinephrine, and epinephrine serum levels in control and treated (cadmium at 5 *μ*g/L during 3 months) specimens. Values are means ± SE of the mean. ^a^Values significantly (*P* < 0.001) different from the control values.

**Figure 2:**
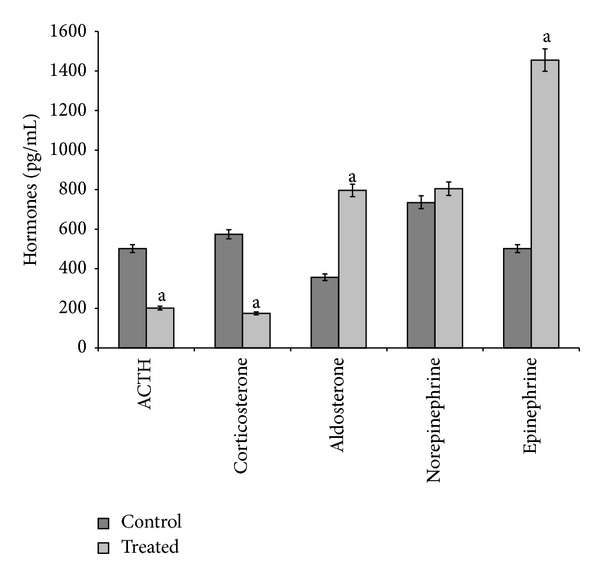
ACTH, corticosterone, aldosterone, norepinephrine, and epinephrine serum levels in control and treated (cadmium at 20 *μ*g/L during 3 months) specimens. Values are means ± SE of the mean. ^a^Values significantly (*P* < 0.001) different from the control values.

During the 3-month exposure, both doses of cadmium (5 *μ*g/L and 20 *μ*g/L) produced the same effects: indeed, they decreased ACTH and corticosterone serum levels, increased aldosterone and epinephrine serum levels, and did not influence norepinephrine serum levels (Figures 1 and 2).

**Figure 3:**
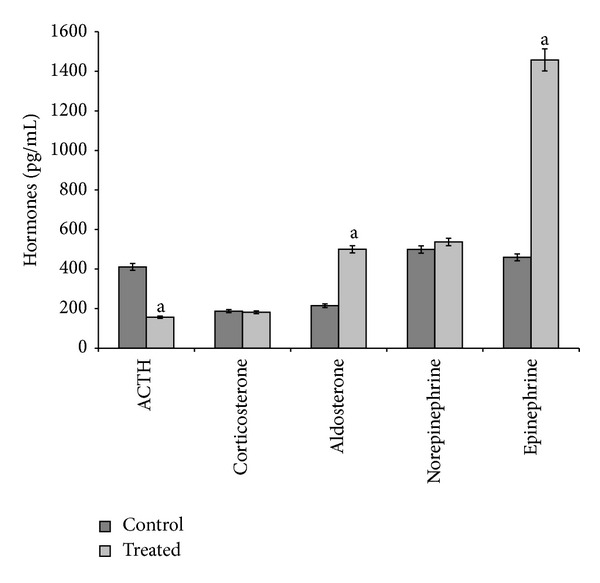
ACTH, corticosterone, aldosterone, norepinephrine, and epinephrine serum levels in control and treated (cadmium at 5 *μ*g/L during 9 months) specimens. Values are means ± SE of the mean. ^a^Values significantly (*P* < 0.001) different from the control values.

**Figure 4:**
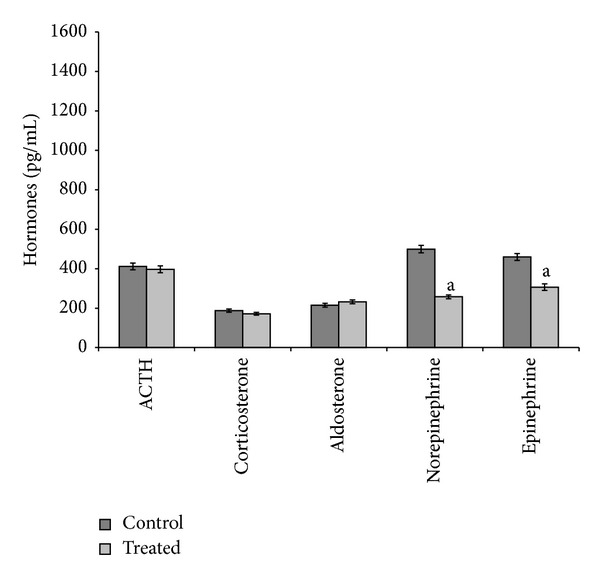
ACTH, corticosterone, aldosterone, norepinephrine, and epinephrine serum levels in control and treated (cadmium at 20 *μ*g/L during 9 months) specimens. Values are means ± SE of the mean. ^a^Values significantly (*P* < 0.001) different from the control values.

During the 9-month exposure, the effects of cadmium depended on the dose administered. At 5 *μ*g/L dose, cadmium decreased ACTH and increased aldosterone and epinephrine serum levels (Figure 3). At 20 *μ*g/L dose, cadmium decreased norepinephrine and epinephrine serum levels, without affecting the other hormones (Figure 4).

## 4. Discussion

Our results show that cadmium, at the freshwater safety values established for this heavy metal by the European Community [30] and the Italian Ministry of Health [31], is able to interfere with the adrenal gland activity of the newt *T. carnifex*, affecting both the steroidogenic and the chromaffin tissues and behaving like an endocrine disruptor. 

Considering the steroidogenic tissue, during the 3-month exposure, both doses (5 and 20 *μ*g/L) of cadmium produced similar effects, decreasing ACTH and corticosterone serum levels and increasing aldosterone serum levels. During the 9-month exposure, the effects of cadmium on the steroidogenic tissue depended on the dose administered. At 5 *μ*g/L dose, cadmium decreased ACTH and increased aldosterone serum levels, without affecting corticosterone levels, whereas at 20 *μ*g/L dose cadmium did not influence the steroidogenic tissue. Our results of the 3-month exposure agree with those of previous studies performed in rats [41], demonstrating an increase in aldosterone and a decrease in corticosterone levels, after treatment with cadmium chloride. The authors suggested that these hormonal variations could be due to an increase in the aldosterone production rate and in the corticosterone metabolic clearance rate, respectively. It is likely that such increases could also take place in the newts. On the other hand, it can be excluded that the cadmium-induced stimulation of aldosterone secretion, observed in the newts, could be mediated through the activation of adrenochromaffin cells since cadmium increased epinephrine serum levels, and, in *T. carnifex*, epinephrine inhibits aldosterone release [42]. Moreover, our results partly agree also with those of recent studies performed in fish, showing that the hypothalamic-pituitary-adrenocortical axis is impaired in fish chronically exposed to cadmium and zinc [28] and that basal and ACTH-stimulated cortisol secretion is compromised by chronic exposure to cadmium [17, 27]. Finally, considering the ACTH levels, the decrease observed in the newts suggests that cadmium may act either at the hypothalamic or pituitary level; moreover, a negative feedback mechanism, caused by the increased aldosterone serum levels, could be hypothesized. 

The analysis of the effects of cadmium on the steroidogenic tissue shows that the same dose produced different effects, depending on the length of exposure. A relatively short period of exposure (3 months) allowed cadmium to interfere, at both 5 and 20 *μ*g/L doses, with the activity of the tissue, probably because the defence mechanisms against these very low cadmium concentrations need more time to be carried out. A longer period of exposure (9 months) probably allowed the organism to carry out these defence mechanisms; indeed, while the lowest dose (5 *μ*g/L) changed only ACTH and aldosterone levels, the highest dose (20 *μ*g/L) did not affect the steroidogenic activity at all. It looks as if, during the 9-month exposure, the lowest dose “passed unobserved” and then was able to interfere with the steroidogenic tissue, whereas the highest dose was blocked by the defence mechanisms of the organism. Metallothioneins, a family of low molecular weight, cysteine-rich proteins, play a key role in cadmium detoxification [43], and their concentration increases after exposure to heavy metals in frogs [20]. It is likely that also in the newt they are involved in the defence mechanisms against cadmium; studies are in progress to evaluate their concentrations during cadmium exposure.

Considering the chromaffin tissue, during the 3-month exposure, both doses of cadmium produced similar effects on the chromaffin tissue, increasing epinephrine serum levels. During the 9-month exposure, the effects of cadmium on the chromaffin tissue depended on the dose administered. At 5 *μ*g/L dose, cadmium increased epinephrine serum levels, as during the 3-month exposure, whereas at 20 *μ*g/L dose cadmium decreased both catecholamines serum levels.

The increase in epinephrine, observed during the 3-month exposure and with the lowest dose during the 9-month exposure, partly agrees with the increase in the levels of adrenal norepinephrine and epinephrine, and in the activity of adrenal tyrosine hydroxylase, observed in rats after chronic cadmium treatment and considered the result of stress induced by this heavy metal [44]. Since in the newts norepinephrine levels were unchanged, and only epinephrine levels were increased, it is likely that cadmium also increased the activity of the enzyme phenylethanolamine-N-methyl transferase (PNMT), methylating norepinephrine into epinephrine, and stimulated epinephrine release, involved, also in amphibians, in neutralizing the stressful stimulus [34]. In contrast, the decrease in the catecholamine levels, observed in the newts during the 9-month exposure at the highest dose (20 *μ*g/L), agrees with the decrease in the baseline concentrations of catecholamines observed in the American eel *Anguilla rostrata*, chronically exposed to cadmium, and considered the result of an interference, cadmium-induced, with catecholamine release from the adrenomedullary equivalent of this fish [45]. 

The analysis of the effects of cadmium on the chromaffin tissue shows that these effects depended on both the dose administered and the length of the exposure. Indeed, whereas the lowest dose (5 *μ*g/L) increased epinephrine release, during the 3- and 9-month exposures, the highest dose (20 *μ*g/L) increased epinephrine release only during the 3-month exposure and decreased both catecholamines release during the 9-month exposure. Further studies are needed to better explain the varied action of cadmium on the chromaffin tissue.

## 5. Conclusion

In conclusion, our results demonstrate that chronic exposure to cadmium, at the safety values established by the European Community and the Italian Ministry of Health, interferes with the adrenal gland activity of the newt, changing the levels of both corticosteroids and catecholamines. Moreover, our results indicate that the effects of cadmium on this gland are related both to the length of exposure and the dose administered. Considering the wide diffusion of cadmium in the aquatic environment, the role of the adrenal gland in the amphibian physiology, and the endocrine-disrupting effect observed in the newt, our results suggest that this metal could contribute to the amphibian decline. Finally, the effects of cadmium on the amphibian adrenal gland suggest probable risks to human health, due to the use of water contaminated by cadmium.
